# Development, Survival and Reproduction of *Spodoptera frugiperda* (Lepidoptera: Noctuidae) Fed an Artificial Diet or on Cotton, Castor Bean and Corn Leaves

**DOI:** 10.3390/insects13050428

**Published:** 2022-05-04

**Authors:** Ruth da Silva Ramos, Carlos Alberto Domingues da Silva, Tardelly de Andrade Lima, Paulo de Souza Albuquerque Junior, Maria Aparecida Castellani, José Eduardo Serrão, José Cola Zanuncio

**Affiliations:** 1 Postgraduate Program in Agricultural Sciences, State University of Paraíba, Campina Grande 58429-500, PB, Brazil; ruthsr01@gmail.com (R.d.S.R.); tardellya@gmail.com (T.d.A.L.); paulo.albuquerque@aluno.uepb.edu.br (P.d.S.A.J.); 2 Entomology Laboratory, Embrapa Cotton, Campina Grande 58428-095, PB, Brazil; 3 Department of Crop Science and Animal Science, State University of Southwestern Bahia, Vitória da Conquista 45083-300, BA, Brazil; castellani@uesb.edu.br; 4 Department of Biology, Federal University of Viçosa, Viçosa 36570-900, MG, Brazil; jeserrao@ufv.br; 5 Department of Entomology/BIOAGRO, Federal University of Viçosa, Viçosa 36570-900, MG, Brazil; zanuncio@ufv.br

**Keywords:** Bionomy, *Gossypium hirsutum*, fall armyworm, *Ricinus communis*, *Zea mays*

## Abstract

**Simple Summary:**

The fall armyworm (Lepidoptera: Noctuidae) is one of the main pests of corn and cotton in South American countries, but it can also feed and survive on castor bean. These plants are cultivated in Brazil in an intercropping and/or succession system in small and large rural properties, at different times of the year near to each other, what can facilitate the movement of this pest and make it difficult to control. The results obtained are promising and confirm our hypothesis that the consumption of host crops less suitable for the fall armyworm can impair its development, survival and reproduction, reducing its infestation in the next host.

**Abstract:**

The polyphagy of *Spodoptera frugiperda* (Lepidoptera: Noctuidae) renders its control difficult because variations in the phenology of host plants grown in different seasons of the year and near each other can facilitate the movement of this pest between crops. The objective of this work was to examine certain biological characteristics of *S. frugiperda* fed on an artificial diet or on cotton, castor bean, or corn leaves. The experimental design was in randomized blocks, with four treatments represented by *S. frugiperda* caterpillars fed an artificial diet (T1) or pieces of cotton (T2), castor bean (T3) or corn (T4) leaves with five replications (five caterpillars per replication). The duration and survival of the egg, larva, pre-pupa, pupa and egg-adult period and the reproductive characteristics of this insect were determined. The survival, development and reproduction data of *S. frugiperda* originated from individuals reared with cotton and castor bean leaves were lower than those fed on corn leaves or an artificial diet. The number of nonviable eggs laid by females originated from caterpillars fed on castor bean leaves was higher than those fed on cotton and corn leaves or an artificial diet. The very higher number of unviable *S. frugiperda* eggs resulting from castor leaves can reduce outbreaks of this pest in cotton and corn crops after the cultivation of that plant, or by intercropping with this plant, in the main producing regions of Brazil.

## 1. Introduction

Cotton (*Gossypium hirsutum* L.), corn (*Zea mays* L.) and castor bean (*Ricinus communis* L.) are important plants cultivated in Brazil. Cotton is the largest source of natural textile fibers in the world [1] and is produced in tropical and subtropical regions [2]. Brazil is the fourth largest producer and second largest exporter of cotton worldwide, mainly to Asian countries [3]. The development of plant material adapted to the soil and climate conditions of the producing regions, and germplasm with the technical characteristics of the fibers required by the textile industry, improved the quality and productivity of Brazilian cotton [4].

Corn, with approximately 1.2 billion tons produced in the 2020/2021 crop globally, had the USA as the world’s largest producer, followed by Brazil [3]. The area planted with corn, for human and animal food, in Brazil was 20.9 million ha in the 2020/2021 harvest, with 117.2 million tons produced and an average productivity of 5596 kg ha^−1^ [5].

Castor bean, a tropical oilseed from the Euphorbiaceae family, is important for the production of oil and the residue (pie) of its fruits used as animal food. This plant is strategic for Brazil [6], the fourth largest producer in the world, with 92% of its production in Bahia State [5].

The fall armyworm, *Spodoptera frugiperda* (J.E. Smith) (Lepidoptera: Noctuidae), one of the main corn and cotton pest in South American countries, including Brazil [7,8], is polyphagous. This insect feeds and survives on castor bean leaves [9,10], although *Spodoptera cosmioides* (Walker, 1858), *Spodoptera latifascia* (Walker, 1856) and *Spodoptera litura* (Fabricius, 1775) are the only pests of this genus reported for this Euphorbiaceae [11,12]. *Spodoptera frugiperda* can have several generations per year with high reproductive and dispersal capacity infesting crops of different plants, including corn and cotton [13].

Variations in the phenology of possible host plants, grown in different seasons of the year near each other, facilitate movement and hamper control of *S. frugiperda* [14]. The development, survival, reproduction and longevity of insects vary according to the host plant [15,16]. This increases the importance of studying *S. frugiperda* in different host plants as greater plant availability affects the population dispersion of this pest [17,18,19].

The biology of *S. frugiperda* has been studied in several plant species [14,20,21], but the characteristics of this pest fed on cotton, castor bean or corn leaves have not been investigated. This is important, as these plants are cultivated in Brazil in an intercropping and/or succession system in small and large rural properties, respectively. Extensive areas of the Cerrado biome in Mato Grosso State previously cultivated with soybean are subsequently cultivated with cotton or corn in a double-cropping system, or left fallow between late February and early March [22]. However, these three plants are generally cultivated using intercropping, mainly on small rural properties in Bahia, Minas Gerais, Piauí and São Paulo states, Brazil [23,24,25]. Castor bean grows in drier conditions with low susceptibility to insect pests and, therefore, can add economic returns without competing with the main crop [22]. Therefore, we hypothesized that the consumption of less suitable host crops for the fall armyworm can impair its development, survival and reproduction, reducing its infestation in the next host.

The objective of this research was to evaluate the development, survival and reproduction of *Spodoptera frugiperda* (Lepidoptera: Noctuidae) fed with an artificial diet and on cotton, castor bean and corn leaves.

## 2. Materials and Methods

### 2.1. Study Location

This study was carried out in the field (7°13′31″ S latitude and 35°54′18″ W longitude) and in the entomology laboratory (7°13′32″ S latitude and 35°54′19″ W longitude) at Embrapa Algodão in the municipality of Campina Grande, Paraíba, Brazil. The soil of the experimental area is classified as Eutrophic Regolithic Neosol [26].

### 2.2. Insects and Plant Material

*Spodoptera frugiperda* specimens were obtained from insect colonies of the entomology laboratory at Embrapa Algodão, where caterpillars of this insect are fed an artificial diet based on wheat germ and casein [27]. The cotton seeds, cultivar BRS 286, and castor beans, cultivar BRS Paraguaçu, were obtained from the Active Germplasm Bank of Embrapa Algodão and the corn seeds, cultivar BRS 3046, acquired through the agricultural products trade. These cultivars were chosen because they are widely used by small and large rural producers in Brazil [28].

### 2.3. Biology Bioassays

Cotton, castor bean and corn seeds were planted in the field with each plant in an area of 60 m^2^ (12 m × 5 m) at Embrapa Algodão in May 2020. Cotton and corn were grown at a spacing of 0.9 m × 0.1 m and the castor bean at 2 m × 1 m between rows and plants, respectively. Fertilization was performed according to the soil analysis and following the technical recommendations for each crop [29]. The fertilizers used were urea (45% N), phosphorus pentoxide (18% P_2_O_5_) and potassium chloride (60% K_2_O) as sources of N-P-K (Fertilizantes Heringer SA, Paulínia, São Paulo, Brazil), respectively.

Two hundred and fifty *S. frugiperda* eggs were obtained from an insect colony and transferred to a Petri dish (10 cm × 0.9 cm in diameter and height, respectively) lined with filter paper until hatching. The newly emerged caterpillars were separated into groups of 50 per treatment and individualized in plastic trays with 16 wells (each well 5.5 cm long × 2.5 cm wide × 2.5 cm high). Wells with larvae were checked daily for larva head capsule exuviae indicating molting to establish the duration of the different larval instars. The number of instars and the respective growth rates (GR) of this insect were determined using the mathematical model based on Dyar [30], as recommended by Parra and Haddad [31]. *Spodoptera frugiperda* caterpillars were immobilized with surgical forceps and brushes and their cephalic capsules measured with the aid of a digital caliper INOX 0–100 mm (Lotus Plus) 24 h after ecdysis.

The experimental design was in randomized blocks, with the four treatments randomly distributed in each block and five replications. The treatments were *S. frugiperda* caterpillars fed on an artificial diet (T1) or pieces of cotton (T2), castor bean (T3) and corn (T4) leaves. Each replication was performed using 10 individual larvae (a total of 50 larvae per treatment = 5 replications of 10 larvae each). The blocks were considered as the different shelves of the acclimatized chamber to reduce the effect of any possible internal temperature gradient from top to bottom. A portion of the artificial diet or a piece of plant leaf were provided every two days for the caterpillar through a plastic well covered with a plastic plate of the same dimensions, and placed in an acclimatized chamber (25 ± 2 °C, 70 ± 10% relative humidity and photoperiod 12 h) until the end of observation. The experiment started when the plants had 9–10 completely expanded leaves with leaf pieces being cut daily from the top of each host plant. Plant leaves were cleaned by immersion in sodium hypochlorite (5%) and rinsed in distilled water for 5 s, and the excess water was removed with paper towels before the leaves were offered to the insects.

The duration and survival of the larva, pre-pupa and pupa period and reproductive characteristics (pre-oviposition, oviposition and post-oviposition periods, longevity and incubation periods, fecundity, number of egg masses, eggs per egg mass and viable eggs) of *S. frugiperda* were determined. The pupae of this insect were weighed on an AY220 analytical balance (Shimadzu Corporation, Columbia, MD, USA) with a precision of 0.0001 g, sexed and kept in PVC cages until adult emergence. The sex ratio was calculated as the division of the number of females by the total number of males and females [32].

Five pairs of *S. frugiperda* were selected and individualized in PVC cages measuring 10 cm × 15 cm (diameter and height), lined internally with continuous paper, as a substrate for oviposition, fed with a 10% honey solution, and kept under the same conditions described above for mating and oviposition until the death of the females. The duration and viability of the egg were determined by separating, the mass laying of each treatment according to the method of Gross et al. [33]. The number of eggs deposited was quantified daily from 2:00 P.M. with the aid of an EL224 stereomicroscope (BEL Engenharia, Monza, Milan, Italy) with 20× magnification. These eggs, obtained daily, were incubated in Petri dishes containing a piece of filter paper moistened with distilled water and the embryonic development was monitored until the moment of hatching to determine the fertility and sex ratio of the *S. frugiperda*. The experiment was carried out in duplicate.

### 2.4. Data Analysis

The percentage survival data were transformed by arcsine of the square root to improve normality before analysis. Data on immature stage duration, pupa weight, head capsule width, growth rate and reproductive characteristics of *S. frugiperda* were transformed using square root to improve normality prior to analysis. Normally distributed data were not transformed. Data were analyzed by ANOVA at *p* ≤ 0.05. Mean significance was separated by the Tukey test. Statistical analyses were performed using the statistical and genetic analysis system (SAEG) [34].

## 3. Results

Survival per *S. frugiperda* instar was higher in the first one with the artificial diet (F_3.12_ = 4.08; *p* < 0.03), second with corn and the artificial diet (F = 3.14; df = 3.12; *p* = 0.05), third (F = 11.02; df = 3.12; *p* < 0.01) and sixth (F = 3.83; df = 3.12; *p* < 0.05) with castor, corn and the artificial diet (Table 1) and similar for the fourth (F= 1.38; df = 3.12; *p* > 0.05), fifth (F= 2.67; df = 3.12; *p* > 0.05) and seventh (F= 3.48; df = 1.4; *p* > 0.05) instars with all diets. *Spodoptera frugiperda* larval stage survival was higher with the artificial diet and corn than with cotton and castor bean leaves (Table 1). The survival of the *S. frugiperda* prepupae and pupae did not differ between the four diets, with values higher than 93% and 78%, respectively (Table 1).

The number of *S. frugiperda* instars varied with the diet (Table 2), with six for caterpillars fed an artificial diet or castor bean and seven with cotton or corn leaves. The duration of the first (F = 35.45; df = 3.12; *p* < 0.01), second (F = 11.55; df = 3.12; *p* < 0.01), third (F = 40.53; df = 3.12; *p* < 0.01), fourth (F = 7.46; df = 3.12; *p* < 0.01), fifth (F = 99.48; df = 3.12; *p* < 0.01), sixth (F = 22.76; df = 3.12; *p* <0.01) and seventh (F = 6.55; df = 1.4; *p* < 0.05) instars and of the larval stage (F = 43.15; df = 3.12; *p* < 0.01) of *S. frugiperda* varied according to diet (Table 2). These periods were shorter for larvae of first, second, third, fourth, fifth and sixth instars of *S. frugiperda* with the artificial diet. The period of the other instars, except for the third and sixth instars of *S. frugiperda* with cotton and the second with castor bean, were always longer with cotton and castor bean leaves. The duration of the larval and pupal stages was longer for *S. frugiperda* fed on cotton than on the artificial diet and castor bean, respectively (Table 2).

The width of the head capsule (HC) and the larva growth ratio (GR) of the first (F = 1.25; df = 3.87; *p* = 0.29), second (F = 7.96; df = 3.79; *p* < 0.01), third (F = 18.13; df = 3.84; *p* < 0.01), fourth (F = 19.99; df = 3.90; *p*< 0.01), fifth (F = 23.00; df = 3.85; *p* < 0.01), sixth (F = 69.19; df = 3.81; *p* < 0.01) and seventh (F = 1.86; df = 1.17; *p* = 0.19) instars of *S. frugiperda* varied according to diet, from 0.24 mm to 2.6 mm, and 1.41 to 1.81, respectively (Table 3). Head capsule width (HC) and growth ratio (GR) of *S. frugiperda* instars were higher with the artificial diet and lower with cotton leaves (Table 3), except for the HC of the seventh and the GR of the fifth and seventh instars with this Malvaceae. The HC of seventh instar *S. frugiperda* larvae with cotton was similar to that with corn and the GR of fifth and seventh instars with cotton was similar and higher than caterpillars fed with the other diets and corn, respectively (Table 3).

Body length (F = 25.71; df = 3.92; *p* < 0.01) and weight (F = 32.44; df = 3.92; *p* < 0.01) of *S. frugiperda* pupae varied between treatments (Table 4). The length of pupae originated from caterpillars fed with an artificial diet was greater than for those fed on cotton, castor bean or corn. The weight of pupae originated from caterpillars fed with corn or an artificial diet was greater than that of those originating from cotton- or castor-bean-fed caterpillars. The sex ratio was similar between treatments.

The oviposition (F = 6.58; df = 3.26; *p* < 0.01) and post-oviposition (F = 3.14; df = 3.26; *p* = 0.04) periods, female (F = 4.60; df = 3.26; *p* = 0.01) and male (F = 11.01; df = 3.26; *p* < 0.01) longevity, egg incubation (F = 15.38; df = 3.78; *p* < 0.01), fecundity (F = 10.03; df = 3.23; *p* < 0.01), number of eggs per egg mass (F = 4.93; df = 3.23; *p* < 0.01) and viable eggs (F = 14.98; df = 3.23; *p* < 0.01) of *S. frugiperda* varied (Table 5), but the pre-oviposition period (F = 1.75; df = 3.26; *p* = 0.18) and the number of eggs laid per female (F = 2.67; df = 3.23; *p* = 0.07) were similar between treatments. The oviposition and post-oviposition periods, fecundity, number of eggs per posture and viable eggs were higher for *S. frugiperda* females originating from caterpillars fed on an artificial diet, followed by those with corn and lowest with castor bean (Table 5).

The longevity of *S. frugiperda* females and males was greater for adults that originated from caterpillars fed with an artificial diet and lower for those that originated from caterpillars fed with leaves of the three plants. The incubation period was shorter for *S. frugiperda* eggs that originated from caterpillars fed an artificial diet and longer for those fed with cotton, castor bean or corn leaves. The pre-oviposition periods and the number of eggs per female did not differ between treatments.

## 4. Discussion

The higher survival rate of *S. frugiperda* first-instar caterpillars on an artificial diet is probably due to the better nutritional quality of this diet and also to the less developed mouthparts of the caterpillars, better adapted to consume soft-textured diets [35,36]. The artificial diet was developed to meet the nutritional requirements of *S. frugiperda* [37]. From the second instar onwards, however, caterpillars are less selective in terms of feeding, eating large amounts of leaf tissue anywhere on the leaf [38], including gossypol glands and ricinin, present in the cotton and castor bean leaves, respectively [39,40,41]. This explains the lower survival of the first-, second- and third-instar caterpillars with cotton, and those of the second instars with castor bean, as these early instars are more sensitive to ingesting gossypol and ricinin, which are present, respectively, in cotton and castor bean leaves. Additionally, the more limited set of digestive enzymes [42] makes them more vulnerable to compounds from these plants [35,43]. However, from the fourth instar onwards, the digestive system of the caterpillars is more developed and, therefore, less vulnerable to toxic metabolites from the host plant leaves [44]. This explains the greater caterpillar survival at more advanced instars with castor leaves, but not for those of the sixth instar with cotton leaves. Caterpillars of this instar fed on cotton leaves were, even with a more developed digestive system, negatively affected by gossypol, which has a cumulative effect on the caterpillars of this genus [45].

The greater survival of the larval stage of *S. frugiperda* with an artificial diet and corn is probably due to the better nutritional quality of these diets (natural and artificial) for this caterpillar [36]. The artificial diet fully meets the nutritional requirements of *S. frugiperda* and corn is one of the main host plants for this insect [37,46]. On the other hand, the lower survival rate of the larval stage of *S. frugiperda* with cotton leaves, followed by those with castor bean, can be attributed, respectively, to the ingestion of gossypol and ricinin, with toxic effects, especially in the initial instars of generalist caterpillars such as *S. frugiperda* [39,40,41]. Survival of *S. frugiperda* caterpillars with cotton and corn leaves was lower than that of 72% and 98% with leaves of the Acala 90 cotton and BRS catingueiro corn [18] and 89% and 91% with leaves of cotton Shyuan 321 and corn Zhengdan 958 [21], respectively, at similar temperatures. This indicates that variations in survival are due to differences between the cotton and corn cultivars, as the nutritional quality varies between species [47] and varieties [48] of host plants.

The high survival of *S. frugiperda* pre-pupae and pupae that originated from caterpillars fed with castor bean, corn and cotton leaves is due to the reduced effect of ricinin, hydroxamic acid (HA) and gossypol, the main secondary metabolites in the leaves of these plants [39,41,49]. The pupal survival of *S. frugiperda* with leaves, respectively, of cotton and corn, was lower and higher than the 89% and 91% with leaves of these plants for this insect at similar temperatures [21], which may be attributed to the differences between the cotton and corn cultivars used.

The lower number of instars with the artificial diet or castor bean (six) than with cotton or corn (seven) of the *S. frugiperda* caterpillars may indicate a compensatory action, when those receiving nutritionally poorer food tend to compensate by increasing the duration of their immature stage [14]. The number of *S. frugiperda* instars varies from five to ten, with the highest number in less suitable host plants and at lower temperatures throughout the geographic distribution of this insect [50,51]. This may be related to the biological plasticity of *S. frugiperda* increasing its chances of development and survival in adverse conditions [52]. Therefore, the additional instars of *S. frugiperda* caterpillars with cotton or corn leaves can be attributed to the physiological or nutritional deficiencies of these plants, which, associated with its high survival rate, suggests a greater adaptive capacity of this insect compared to other species of this genus [51].

The shortest periods of the first, second, third, fourth and fifth instars of *S. frugiperda* with the artificial diet and the longest periods of the first, second, fourth, fifth and seventh instars with cotton leaves and the first, third, fourth, fifth and sixth instars with castor bean leaves can be attributed, as mentioned, to the ingestion of gossypol and ricinin from these plants. These compounds, present in cotton and castor beans, may have reduced the growth and food conversion efficiency of the *S. frugiperda* caterpillars [40,53,54].

The longer duration of the larva and pupa stages of *S. frugiperda* with cotton seems to indicate that the leaves of this plant are nutritionally less adequate than those of castor bean and corn. However, the additional instar with cotton leaves and corn indicates adverse conditions when larvae cannot reach a specific size threshold for metamorphosis [52] because of the cumulative toxic effect of gossypol and hydroxamic acid (HA) in cotton and corn leaves, respectively [39,48]. The duration of the larval stage of *S. frugiperda* with cotton leaves was longer than the 18 [14], 21 [18] and 21 [21] days with cotton leaves of FMT 701, Acala 90 and Shiyuan 321, respectively, at similar temperatures. On the other hand, the duration of the larval stage, with corn leaves, was similar to the 16 days [14] with corn leaves DKB 360 and greater than the 13.7 [18] and 14.5 [21] days with the BRS Caatingueiro and Zhengdan 958 corn leaves, respectively, at a similar temperature. These variations are due to the differences between the cotton and corn cultivars used. This also explains the similar duration of the pupa stage of *S. frugiperda* with cotton leaves to the 11 days [21] with the Zhengdan 958 cotton leaves and greater than the 9.5 [18] and 9.4 [14] days with the Acala 90 and FMT 701 cotton leaves, respectively; and greater than the 9.2 [18], 9.0 [21], and 8.5 [14] days with the BRS Caatingueiro, Zhengdan 958 and DKB 360 corn leaves, respectively, at a similar temperature.

Variations in the head capsule width and growth rate of the *S. frugiperda* caterpillars fed on cotton, castor bean or corn leaves, and an artificial diet were similar to the pattern for the head capsule of this insect [51,55] with a growth rate ratio of 1.1 to 1.9 as established by Dyar’s rule [30]. The largest widths of the head capsule (HC) and the growth ratio (GR) of *S. frugiperda* instars with the artificial diet and the smallest with cotton leaves indicate a negative effect of gossypol as demonstrated on *Spodoptera littoralis* (Lepidoptera: Noctuidae) with leaves of this plant [56,57]. This demonstrates the importance of knowing the nutritional value of host plants for the development of management practices aimed at minimizing the severity of damage by polyphagous insects such as *S. frugiperda* [58,59].

The greater length and weight of pupae that originated from the *S. frugiperda* caterpillars fed an artificial diet (without toxic metabolites) than those fed on cotton or castor bean leaves indicate that the toxic effect of the metabolites from the leaves of these plants reduces food conversion and absorption of essential nutrients by this insect to form its pupae. This is important as the length and weight of pupae are correlated with female fertility as demonstrated for *Spodoptera eridania* (Stoll, 1782) (Lepidoptera: Noctuidae) pupae that originated from caterpillars fed an artificial diet [60,61].

The longer oviposition and post-oviposition periods, fecundity, number of eggs per egg mass, and viable eggs of *S. frugiperda* females that originated from caterpillars fed on an artificial diet and corn leaves, than from those caterpillars fed on castor and cotton leaves indicates that the artificial diet and corn leaves are more suitable for this insect. The better outcomes from the artificial diet and corn leaves for the rearing, development and reproduction of *S. frugiperda* is due to their composition, in carbohydrates, amino acids, trace elements, fatty acids, vitamins and water [21,62,63]. On the other hand, the shorter oviposition and incubation periods, better fecundity and higher number of nonviable eggs of *S. frugiperda* females that originated from caterpillars fed on cotton and castor bean leaves indicates that the gossypol and ricinin content of these leaves, respectively, are harmful to these reproductive parameters of this insect [40,53,54]. The high number of nonviable eggs of *S. frugiperda* females that originated from caterpillars fed on castor bean leaves is probably due to the ingestion of ricinin, an alkaloid with repellent and toxic properties for caterpillars of this insect [40,64]. This indicates that the consumption of palatable and poorly nutritious plant species, such as castor bean leaves, can reduce *S. frugiperda* populations. However, the shorter pre-oviposition period of *S. frugiperda* that originated from caterpillars fed on cotton and corn leaves, than the 3.7 and 3.9 days for this insect on the IAC 17 cotton leaves [65] and CO6 corn leaves [66], under similar temperature and relative humidity, indicates the effect of cotton cultivars on these parameters. This may explain the shorter oviposition period of *S. frugiperda* females that originated from caterpillars fed with corn leaves than the 6.1 days of this insect with the CO6 corn [66], under the same conditions, and also the similar longevity but lower number of females of this insect that originated from caterpillars fed on cotton leaves and corn, respectively, than the nine days from the IAC 17 cotton leaves [65] and the 12.6 days with the CO6 corn [66] for this insect, under the same conditions. This also explains the shorter longevity of males with cotton and corn leaves, than the nine days with the IAC 17 cotton [65] and the 11.1 days with the corn CO6 [66]. In addition, the lower number of eggs per *S. frugiperda* female resulting from caterpillars fed on cotton leaves than the 774 eggs per female with the IAC 17 cotton [65] and greater than the 427.3 eggs per female with the CO6 corn [66] confirms the effect of the cotton and corn cultivars used, with different nutritional quality between species and biotypes of the host plants.

The lower survival, development and reproduction values of *S. frugiperda* that originated from caterpillars fed on cotton and castor bean leaves, and the high number of unviable eggs per female originated from those fed on castor bean leaves, may be important for the management of this pest. *Spodoptera frugiperda* females oviposit on several plant species, including the most abundant and the least common, mainly in the absence of preferred hosts [9]. Therefore, castor bean as a main crop or intercropping with cotton or corn can reduce oviposition and development of immature stages of *S. frugiperda*. The females of this population will deposit more unviable eggs in subsequent crops (cotton and corn) after the castor bean crop or when intercropping with it, which can reduce population outbreaks of this pest.

## Figures and Tables

**Table 1:** Survival (%, mean ± standard error) of the larva stage and of first, second, third, fourth, fifth, sixth and seventh instars, pre-pupa and pupa of *Spodoptera frugiperda* (Lepidoptera: Noctuidae) fed with pieces of cotton (*Gossypium hirsutum* L.), castor bean (*Ricinus communis* L.), and corn (*Zea mays* L.) leaves an artificial diet at 25 ± 2 °C, relative humidity of 60 ± 10 and photophase of 12 h.

Stage	Instar	Cotton	Castor Bean	Corn	Artificial Diet
Larval		42.00 ± 9.70 b	66.00 ± 7.48 ab	80.00 ± 4.47 a	86.00 ± 6.00 a
	First	78.00 ± 8.00 b	84.00 ± 4.00 ab	88.00 ± 3.74 ab	98.00 ± 2.00 a
	Second	87.50 ± 6.18 b	87.62 ± 5.78 b	100.00 ± 0.00 a	94.00 ± 4.00 ab
	Third	81.62 ± 3.02 b	93.14 ± 4.30 a	100.00 ± 0.00 a	100.00 ± 0.00 a
	Fourth	100.00 ± 0.00 a	97.50 ± 2.50 a	100.00 ± 0.00 a	93.33 ± 4.44 a
	Fifth	100.00 ± 0.00 a	100.00 ± 0.00 a	95.00 ± 3.06 a	100.00 ± 0.00 a
	Sexth	83.00 ± 7.68 b	97.78 ± 2.22 a	100.00 ± 0.00 a	100.00 ± 0.00 a
	Seventh	88.78 ± 5.09 a	-	80.13 ± 5.97 a	-
Pre-pupa		93.33 ± 6.67 a	100.00 ± 0.00 a	100.00 ± 0.00 a	100.00 ± 0.00 a
Pupa		78.89 ± 9.69 a	100.00 ± 0.00 a	97.78 ± 2.22 a	95.00 ± 5.00 a

Means followed by the same letter in the line do not differ by the Tukey test (*p* ≤ 0.05). Means of survival percentages of immature stages of *S. frugiperda* analyzed were transformed into the arcsine of the square root for statistical analysis. Original averages shown. (-) No data.

**Table 2:** Developmental period (days, mean ± standard error) of the larvae stage and of the first, second, third, fourth, fifth, sixth and seventh instars, pre-pupa and pupa of *Spodoptera frugiperda* (Lepidoptera: Noctuidae) fed with pieces of cotton (*Gossypium hirsutum* L.), castor bean (*Ricinus communis* L.) and corn (*Zea mays* L.) leaves and an artificial diet at 25 ± 2 °C, relative humidity of 60 ± 10 and photophase of 12 h.

Stages	Instar	Cotton	Castor Bean	Corn	Artificial Diet
Larval		25.18 ± 1.41 a	21.43 ± 0.59 b	16.73 ± 0.26 c	12.41 ± 0.23 d
	First	3.19 ± 0.10 a	3.19 ± 0.07 a	2.65 ± 0.13 b	2.00 ± 0.00 c
	Second	2.79 ± 0.11 a	2.07 ± 0.22 b	1.53 ± 0.14 b	1.83 ± 0.08 b
	Third	2.41 ± 0.08 b	3.00 ± 0.20 a	1.67 ± 0.14 c	1.12 ± 0.07 d
	Fourth	2.90 ± 0.22 a	3.24 ± 0.32 a	2.68 ± 0.11 ab	1.73 ± 0.07 b
	Fifth	4.27 ± 0.17 a	3.83 ± 0.10 a	2.54 ± 0.07 b	1.90 ± 0.06 c
	Sixth	4.83 ± 0.41 b	6.17 ± 0.36 a	2.74 ± 0.15 c	3.83 ± 0.12 bc
	Seventh	5.33 ± 0.84 a	-	3.20 ± 0.23 b	-
Pre-pupa		1.25 ± 0.19 a	1.33 ± 0.04 a	1.21 ± 0.10 a	1.09 ± 0.06 a
Pupa		11.49 ± 0.16 a	9.00 ± 0.00 d	9.88 ± 0.09 c	10.66 ± 0.11 b

Means followed by the same letter in the line do not differ by the Tukey test (*p*≤ 0.05). (-) No data.

**Table 3:** Head capsule (HC) width and growth ratio (GR) of the first, second, third, fourth, fifth, sixth and seventh instars of *Spodoptera frugiperda* (Lepidoptera: Noctuidae) fed with pieces of cotton (*Gossypium hirsutum* L.), castor bean (*Ricinus communis* L.) and corn (*Zea mays* L.) leaves and an artificial diet at 25 ± 2 °C, relative humidity of 60 ± 10 and photophase of 12 h.

Larva Instar	Variable	Cotton	Castor Bean	Corn	Artificial Diet
First	HC	0.30 ± 0.00 a	0.30 ± 0.00 a	0.29 ± 0.00 a	0.30 ± 0.00 a
	GC	-	-	-	-
Second	HC	0.43 ± 0.01 b	0.43 ± 0.00 b	0.43 ± 0.00 b	0.46 ± 0.01 a
	GC	1.41 ± 0.03 b	1.46 ± 0.03 ab	1.48 ± 0.01 ab	1.57 ± 0.04 a
Third	HC	0.71 ± 0.01 c	0.75 ± 0.01 b	0.80 ± 0.01 a	0.82 ± 0.01 a
	GC	1.67 ± 0.03 b	1.77 ± 0.04 ab	1.81 ± 0.05 a	1.80 ± 0.02 a
Fourth	HC	1.05 ± 0.02 b	1.25 ± 0.03 a	1.21 ± 0.02 a	1.29 ± 0.01 a
	GC	1.48 ± 0.03 b	1.67 ± 0.05 a	1.51 ± 0.02 b	1.59 ± 0.02 ab
Fifth	HC	1.52 ± 0.03 c	1.73 ± 0.03 b	1.72 ± 0.03 b	1.87± 0.01 a
	GC	1.45 ± 0.02 a	1.44 ± 0.04 a	1.42 ± 0.02 a	1.47 ± 0.02 a
Sixth	HC	1.97 ± 0.07 d	2.45 ± 0.03 b	2.15 ± 0.01 c	2.63 ± 0.02 a
	GC	1.30 ± 0.03 b	1.42 ± 0.02 a	1.28 ± 0.02 b	1.42 ± 0.01 a
Seventh	HC	2.59 ± 0.05 a	-	2.57 ± 0.02 a	-
	GC	1.37 ± 0.08 a	-	1.20 ± 0.01 b	-

Means followed by the same letter in the line do not differ by the Tukey test (*p*≤ 0.05). (-) No data.

**Table 4:** Pupae body length and weight and sex ratio (mean ± standard error) of *Spodoptera frugiperda* (Lepidoptera: Noctuidae) fed on cotton (*Gossypium hirsutum* L.), castor bean (*Ricinus communis* L.), corn (*Zea mays* L.) and an artificial diet at 25 ± 2 °C, relative humidity of 60 ± 10 and photophase of 12 h.

Variable	Cotton	Castor Bean	Corn	Artificial Diet
Length (mm)	13.88 ± 0.11 c	14.25 ± 0.14 bc	14.88 ± 0.17 b	15.48 ± 0.14 a
Weight (g)	0.14 ± 0.00 b	0.15 ± 0.00 b	0.18 ± 0.01 a	0.19 ± 0.00 a
Sex ratio	0.45	0.53	0.55	0.49

Means followed by the same letter in the line do not differ by the Tukey test (*p*≤ 0.05).

**Table 5:** Reproductive parameters (mean ± standard error) of *Spodoptera frugiperda* (Lepidoptera: Noctuidae) fed with pieces of cotton (*Gossypium hirsutum* L.), castor bean (*Ricinus communis* L.), corn (*Zea mays* L.) leaves and an artificial diet at 25 ± 2 °C, relative humidity of 60 ± 10 and photophase of 12 h.

Parameters	Treatments			
	Cotton	Castor Bean	Corn	Artificial Diet
Pre-ovip. (days)	3.00 ± 0.49 a^(1)^	3.70 ± 0.79 a	2.11 ± 0.20 a	2.10 ± 0.18 a
Ovip. (days)	2.50 ± 0.60 b	2.40 ± 0.52 b	4.67 ± 0.55 a	5.50 ± 0.65 a
Post-ovip. (days)	0.90 ± 0.23 ab	0.90 ± 0.41 b	1.00 ± 0.17 ab	2.20 ± 0.47 a
Fem. long. (days)	9.30 ± 0.58 ab	9.10 ± 0.60 ab	7.89 ± 0.42 b	11.50 ± 0.85 a
Male long. (days)	7.20 ± 0.53 b	5.80 ± 0.65 b	6.89 ± 0.45 b	9.40 ± 0.31 a
Inc. per. (days)	3.29 ± 0.11 ab	4.50 ± 0.50 a	2.96 ± 0.11 b	2.44 ± 0.09 c
No. of eggs per female	312.11 ± 70.65 b	216.63 ± 58.85 b	663.78 ± 110.40 ab	1276.60 ± 157.26 a
No. of laying	7.56 ± 1.79 a	6.00 ± 1.41 a	8.11 ± 1.25 a	12.80 ± 0.74 a
No. eggs per laying	50.00 ± 12.43 ab	40.18 ± 10.78 b	80.27 ± 8.22 ab	96.00 ± 5.58 a
Viable eggs (%)	44.24 ± 12.42 b	3.76 ± 2.46 c	61.70 ± 6.66 ab	80.74 ± 2.37 a

Pre-ovip. = pre-oviposition, Ovip. = oviposition, Post-ovip. = post-oviposition, Fem. Long. = female longevity, Male long. = male longevity, Inc. per. (days) = incubation period, No. of laying = number of egg masses deposited, No. eggs per laying = number of eggs per egg masses. Means followed by the same letter in the line do not differ by the Tukey test (*p* ≤ 0.05). Means of pre-oviposition, oviposition, post-oviposition, number of eggs, postures and eggs per posture of *S. frugiperda* transformed in √x + 0.5 for statistical analysis. Original averages shown.

## Data Availability

Data are contained within the article.
